# Myocardial ischemia during ventilator weaning: a prospective multicenter cohort study

**DOI:** 10.1186/s13054-019-2601-8

**Published:** 2019-09-18

**Authors:** Alexandre Bedet, Françoise Tomberli, Gwenael Prat, Pierre Bailly, Achille Kouatchet, Sater Mortaza, Emmanuel Vivier, Sylvene Rosselli, Larissa Lipskaia, Guillaume Carteaux, Keyvan Razazi, Armand Mekontso Dessap

**Affiliations:** 1 Medical Intensive Care Unit, DHU A-TVB, Henri Mondor University Hospital, Assistance Publique – Hôpitaux de Paris, Créteil, F-94010 France; 2 Mondor Institute of Biomedical Research, CARMAS Research Group, Paris Est Créteil University, Créteil, F-94010 France; 3 Surgical Intensive Care Unit, DHU A-TVB, Henri Mondor University Hospital, Assistance Publique – Hôpitaux de Paris, Créteil, F-94010 France; 4 Intensive Care Unit, Cavale Blanche, Brest Regional University Hospital, 29200 Brest, France; 5 Medical Intensive Care Unit, Angers University Hospital, 49933 Angers, France; 6 Intensive Care Unit, Saint-Joseph Saint-Luc Hospital, 69007 Lyon, France

**Keywords:** Weaning, Cardiac ischemia, Cardiac pulmonary edema, ST monitoring, Echocardiography

## Abstract

**Background:**

Weaning-induced cardiac pulmonary edema (WiPO) is one of the main mechanisms of weaning failure during mechanical ventilation. We hypothesized that weaning-induced cardiac ischemia (WiCI) may contribute to weaning failure from cardiac origin.

**Methods:**

A prospective cohort study of patients mechanically ventilated for at least 24 h who failed a first spontaneous breathing trial (SBT) was conducted in four intensive care units. Patients were explored during a second SBT using multiple tools (echocardiography, continuous 12-lead ST monitoring, biomarkers) to scrutinize the mechanisms of weaning failure. WiPO definition was based on three criteria (echocardiographic signs of increased left atrial pressure, increase in B-type natriuretic peptides, or increase in protein concentration during SBT) according to a conservative definition (at least two criteria) and a liberal definition (at least one criterion). WiCI was diagnosed according to the third universal definition of myocardial infarction proposed by the European Society of Cardiology (ESC) and the American Heart Association (AHA) statement for exercise testing.

**Results:**

Among patients who failed a first SBT, WiPO occurred in 124/208 (59.6%) and 44/208 (21.2%) patients, according to the liberal and conservative definition, respectively. Among patients with ST monitoring, WiCI was diagnosed in 36/177 (20.3%) and 12/177 (6.8%) of them, according to the ESC and AHA definitions, respectively. WiCI was not associated with WiPO and was not associated with weaning outcomes. Only two patients of the cohort were treated for an acute coronary syndrome after the second SBT, and seven other patients required coronary angiography during the weaning period.

**Conclusions:**

This observational study showed the common occurrence of pulmonary edema in mechanically ventilated patients who failed a first SBT, but the association with cardiac ischemia and weaning outcomes was weak.

## Background

The weaning process represents a critical step for patients admitted in intensive care units and requiring mechanical ventilation. Weaning failure is associated with poor outcomes, and prolonged weaning is associated with increased risk of death [1–3]. Standardized weaning protocols with the use of spontaneous breathing trials (SBTs) seem to shorten the duration of mechanical ventilation and could help the clinician to scrutinize the causes of weaning failure [4]. Weaning-induced cardiac pulmonary edema (WiPO), which was described many decades ago [5], is one of the main causes of weaning failure [6]. Recent advances in its diagnosis have been made using new tools such as bedside echocardiography [7] or biomarkers [8–10]. However, the underlying mechanisms of WiPO remain controversial [11]. Weaning-induced cardiac ischemia (WiCI) may be a key factor, as weaning from positive-pressure ventilation induces some physiologic changes that increase cardiac work and myocardial oxygen demand [12]. However, the relationship between WiCI and weaning outcomes is generally poorly described. This study primarily aimed at exploring WiCI and WiPO in patients who failed a first SBT. In these patients, cardiac function was assessed using multiple tools (echocardiography, ST monitoring, and cardiac biomarkers) during a second weaning trial. We hypothesized that WiCI may contribute to WiPO, which may influence weaning outcomes.

## Methods

Guidelines for reporting this study were derived from the Strengthening the Reporting of Observational Studies in Epidemiology (STROBE) Statement [13].

### Study population

This prospective multicenter cohort study was performed in four medical or mixed intensive care units of secondary and tertiary hospitals in France from February 2012 to May 2016. Patients screened for enrolment were those intubated for at least 24 h with ventilator settings allowing to initiate the weaning process [SpO_2_ > 90% or PaO_2_/FiO_2_ ≥ 150 mmHg with a fraction of inspired oxygen (FiO_2_) ≤ 40% and a positive end-expiratory pressure (PEEP) ≤ 8 cmH_2_O]. Exclusion criteria included age < 18 years, decision to withdraw life support, hemodynamic instability with significant doses of vasopressors (dopamine or dobutamine > 10 μg/kg/min, epinephrine or norepinephrine > 0.5 mg/h), patient deeply comatose or sedated, and extreme temperatures (< 36 °C or > 39 °C). The modality of the first SBT was either a low-pressure support ventilation without PEEP or a T-piece, as per the recommendations [14, 15] and the usual care in the participating units. Criteria for SBT failure were respiratory rate ≥ 35 breaths/min or increase ≥ 50%, SpO_2_ ≤ 90% or PaO_2_ ≤ 50 mmHg (with FiO_2_ ≥ 50%), heart rate ≥ 140 beats/min, new onset of supraventricular or ventricular arrhythmia, systolic arterial pressure > 180 or < 90 mmHg, alteration of consciousness, and diaphoresis or any signs of respiratory distress [14, 15]. Patients who failed the first SBT were included in the study.

This study was conducted in accordance with the amended Declaration of Helsinki. The protocol was approved by our institution’s local ethics committee (Comité de Protection des Personnes Ile-de-France IX, approval number 10-064). The protocol was considered a component of standard care and the patient’s consent was waived. Written and oral information about the study was given to patients or families.

### Second SBT and weaning outcomes

No specific therapeutic interventions were recommended to the clinician after the failure of the first SBT. A second SBT, consisting of a 2-h T-piece trial [16], was performed within 24 h after the first SBT in all included patients. We chose the T-piece trial for the second SBT because this modality may be more prone to stress the cardiorespiratory system in order to diagnose WIPO [6]. Criteria for second SBT failure were the same as for the first SBT. Patients who succeeded the second SBT were extubated. Successful weaning was defined as patient alive and not reintubated within the 7 days following extubation, irrespective of the use of noninvasive ventilation [15]. We classified patients into three groups, according to the WIND classification [1], as follows: short weaning (successful weaning or death within 1 day after the first SBT), difficult weaning (successful weaning or death after more than 1 day but in less than 7 days after the first SBT), and prolonged weaning (successful weaning or death after 7 days following the first SBT).

### Definition of WiPO

Because there is no noninvasive consensual definition of WiPO, we considered three criteria proposed in the recent literature: (i) echocardiographic signs of increased left atrial pressure at the end of the SBT: E/A ratio > 0.95 and E/e’ ratio > 8.5 [17]; (ii) an increase of BNP (absolute change ≥ 48 ng/l) or NT-proBNP (absolute change ≥21 ng/l) concentration during the SBT [9]; (iii) an increase of protein concentration (relative change > 6%) during the SBT [8]. We further combined these criteria into two definitions of WiPO, as follows: a conservative definition (when at least two criteria were fulfilled) and a liberal definition (when only one criterion was fulfilled).

### Echocardiography

Transthoracic echocardiography was performed by a trained operator just before and at the end of the second SBT, as previously described [18]. Briefly, left ventricular ejection fraction was assessed using Simpson’s biplane method or estimated visually when poor spatial resolution did not allow sufficient identification of the endocardium. Wall motion analysis was also visually assessed [19]. In the apical four-chamber view, left atrial pressure were estimated by assessing early (E) and late (A) diastolic wave velocities at the mitral valve using the pulsed-wave Doppler; tissue Doppler early (e’) and late (a’) wave velocities were also assessed at the lateral mitral valve annulus. Pulsed-wave Doppler of the left ventricular outflow tract was assessed in the apical five-chamber view for cardiac output computation. The existence of significant (at least moderate) mitral regurgitation was assessed using color Doppler [20].

### Biomarkers

During second SBT, venous samples were collected to measure plasma levels of brain natriuretic peptide (BNP) or amino terminal pro-brain natriuretic peptide (NT-proBNP), protein, and high-sensitive cardiac troponin T (or troponin I in non-equipped centers) at three time points: before the SBT (in all patients), at the end of SBT (in all patients, whether it was a success or a failure), and 2 h after the start of SBT (only in patients reconnected prematurely to the ventilator because of SBT failure). We also assessed arterial blood gas analyses before and at the end of SBT.

### Definition of WiCI

ST segment measurements were recorded every minute during the second SBT using a continuous 12-lead electrocardiogram via the monitoring station. We adapted the third universal definition of myocardial infarction proposed by the European Society of Cardiology (ESC) [21] to define electrocardiographic WiCI, as follows: ST elevation in two contiguous leads (≥ 0.10 mV in all leads other than V_2_–V_3_; ≥ 0.20 mV in V_2_–V_3_ in men ≥ 40 years; ≥ 0.25 mV in V_2_–V_3_ in men < 40 years; ≥ 0.15 mV in V_2_–V_3_ in women), or ST depression ≥ 0.05 mV in two contiguous leads. As weaning shares some similarities with a cardiac stress test, another definition was extrapolated from the American Heart Association (AHA) statement for exercise testing as follows: ST elevation or depression ≥ 0.10 mV in two contiguous leads [22].

### Statistical analysis

We hypothesized that the previously reported WiCI prevalences of 6–10% were probably underestimated given the ancient monitoring techniques used [23, 24]. We estimated that a sample size of 200 patients would allow detecting a prevalence of WiCI of at least 15% with an accuracy of 5% and an alpha risk of 5% (95% confidence interval). The data were analyzed using SPSS Base 20 (IBM-SPSS Inc., Chicago, IL, USA). Categorical variables were expressed as numbers [percentage], continuous data with normal distribution as means [standard deviation], and continuous data with non-normal distribution as medians [25th–75th percentiles]. We used the chi-squared or Fisher exact test to compare categorical variables between groups. The Kruskal-Wallis test was used to compare independent data with non-normal distribution and the Mann-Whitney test to compare paired data. Two-sided *p* values less than 0.05 were considered significant.

## Results

A total of 1749 mechanically ventilated patients were screened for the study. Among these patients, 211 failed a first SBT and 208 were included in the study; ST monitoring was available in 177 of these patients.

### Patient population

Among patients who failed a first SBT, weaning was short in 51/208 [25%], difficult in 95/208 [46%], and prolonged in 62/208 patients [30%] according to the WIND classification. No patients died in the short group while 9/95 patients [9.5%] and 35/62 patients [56.5%] died in the difficult and prolonged group, respectively. Patient characteristics were similar in the three groups except for a higher prevalence of heart failure with reduced ejection fraction, septic shock, and ventilator-associated pneumonia before weaning in the prolonged group, as compared to other groups (Additional file 1). Forty-one over 208 [19.7%] patients had a past history of coronary artery disease, and this prevalence did not differ between the three groups (Additional file 1). The clinical and biological parameters assessed just before the second SBT were also similar between groups, except for longer duration of mechanical ventilation since intubation, higher prevalence of the assist-control ventilation mode and of pulmonary consolidations, more fluid balance since admission, and lower values of MRC score and hemoglobin concentration in patients with poor weaning outcomes as compared to those with short weaning (Table 1). Patients with prolonged weaning had a longer length of stay in ICU and a higher mortality as compared to their counterparts (Additional file 1).

**Table 1:** Clinical and biological data of 208 patients before the second spontaneous breathing trial (SBT)

Clinical and biological data	All patients (*n* = 208)	Weaning outcome			*p*
		Short (*n* = 51)	Difficult (*n* = 95)	Prolonged (*n* = 62)	
Clinical data					
Body weight, kg	78 (66–95)	81 (68–90)	78 (66–96)	78 (65–92)	0.962
Variation of weight since admission, kg	0 (−2 to 4)	0 (−2 to 4)	0 (−1 to 3)	1 (−2 to 5)	0.965
SOFA score	3 (3–5)	3 (2–4)	3 (3–5)	4 (3–6)	0.130
Richmond Agitation-Sedation Score	0 (0–0)	0 (0–0)	0 (0–0)	0 (0–0)	0.230
Temperature, °C	37.8 (37.5–38.2)	37.8 (37.5–38.2)	37.7 (37.3–38.2)	37.7 (37.4–38.1)	0.745
Fluid balance since admission, ml	4500 (1677–10,966)	3471 (737–6498)	4161 (1052–9654)	8653 (2838–15,121)	0.002
Use of diuretics since admission	111 (53.4)	26 (51.0)	48 (50.5)	37 (59.7)	0.492
Spontaneous cough	155 (76.0)	43 (86.0)	69 (73.4)	43 (71.7)	0.130
Delirium (CAM-ICU)	70 (33.6)	16 (31.4)	32 (33.7)	22 (35.5)	0.915
New or persistent radiological pulmonary consolidation	58 (28.3)	14 (28.6)	20 (21.1)	24 (39.3)	0.047
Duration of mechanical ventilation since intubation	6 (4–13)	6 (3–8)	6 (3–9)	13 (7–20)	< 0.001
Mechanical ventilation					0.013
Assist-control ventilation	35 (16.9)	5 (9.8)	13 (13.8)	17 (27.4)	
Pressure support ventilation	170 (82.1)	46 (90.2)	81 (86.2)	43 (69.4)	
Others	2 (1.0)	0 (0.0)	0 (0.0)	2 (3.2)	
Heat and moisture exchanger	141 (68.4)	31 (60.8)	65 (69.9)	45 (72.6)	0.374
Pressure support level, cmH_2_O	12 (10–14)	12 (10–13)	12 (10–14)	12 (10–14)	0.229
PEEP, cmH_2_O	5 (5–6)	5 (5–5)	5 (5–6)	5 (5–5)	0.362
Tidal volume, ml	438 (376–499)	434 (359–479)	443 (380–512)	430 (378–472)	0.553
Tidal volume per kilogram body weight, mL/kg	7.1 (6.2–8.1)	7.0 (6.3–8.3)	7.3 (6.3–8.2)	6.7 (6.1–7.9)	0.453
Respiratory rate, breaths per minute	26 (20–31)	27 (19–32)	25 (21–29)	26 (20–31)	0.804
Number of tracheal suctions during 24 h	7 (5–11)	8 (6–10)	8 (5–12)	6 (4–11)	0.424
MRC muscle scale	50 (9–60)	60 (40–60)	52 (20–60)	25 (5–48)	0.002
Biological data					
White blood count, G/l	11.4 (8.3–15.2)	11.1 (7.9–13.4)	11.8 (8.9–15.1)	12.4 (7.8–16.9)	0.460
Creatinine, micromol/l	78 (53–140)	82 (58–121)	77 (52–142)	75 (52–147)	0.761
Protid, g/l	59 (54–65)	59 (55–66)	57 (53–64)	61 (55–66)	0.224
Hemoglobin, g/dl	9.4 (8.2–10.8)	9.6 (8.3–11.2)	9.6 (8.4–10.8)	9.0 (7.9–10.1)	0.042
Positive lower respiratory tract sample	30	9	10	11	0.341
Time since intubation, days	6 (4–13)	6 (3–8)	6 (3–9)	13 (7–20)	< 0.001
Type of the second SBT					0.455
T-piece	204 (98.1)	51 (100.0)	93 (97.9)	60 (96.8)	
Pressure support ventilation without PEEP	4 (2.0)	0 (0.0)	2 (2.1)	2 (3.2)	
Duration of the second SBT, minutes	60 (17–120)	120 (55–123)	50 (18–120)	24 (10–97)	< 0.001
Failure of the second SBT	132 (63.5)	16 (31.4)	68 (71.6)	48 (77.4)	< 0.001
Reasons for second SBT failure^a^					
Respiratory rate > 35/min	89 (65.4)	10 (58.8)	43 (61.4)	36 (73.5)	0.329
SpO_2_ < 90%	47 (34.6)	6 (35.3)	25 (35.7)	16 (32.7)	0.940
PCO_2_ > 50 mmHg	9 (6.6)	0 (0.0)	8 (11.4)	1 (2.0)	0.064
Heart rate > 140/min	4 (2.9)	0 (0.0)	2 (2.9)	2 (4.1)	0.691
Systolic arterial pressure > 180 mmHg	21 (15.4)	2 (11.8)	10 (14.3)	9 (18.4)	0.752
Increased work of breathing or distress	68 (50.0)	8 (47.1)	33 (47.1)	27 (55.1)	0.671
Alteration of consciousness	6 (4.4)	0 (0.0)	1 (1.4)	5 (10.2)	0.046

*SOFA* Sepsis-related Organ Failure Assessment, *CAM-ICU* Confusion Assessment Method for the Intensive Care Unit, *PEEP* positive end-expiratory pressure, *MRC* Medical Research Council, *SBT* spontaneous breathing trial

Data are expressed as number (percentage) for categorical variables or median (1st quartile–3rd quartile) for continuous variables

Weaning outcome was defined as follows: short weaning (successful weaning or death within 1 day after the first SBT), difficult weaning (successful weaning or death after more than 1 day but in less than 7 days after the first SBT), and prolonged weaning (successful weaning or death after 7 days following the first SBT)

^a^132 patients failed the second spontaneous breathing trial

### Second SBT

The median delay between the first and second SBT was 1 day [0–1]. One hundred and thirty-two over 208 patients (63.5%) failed the second SBT (Table 1). During the second SBT, NT-pro BNP and echocardiographic surrogates of left atrial pressure (E, E/A, and E/e’) increased in the failure group, but not in the success group, while an increase in protein concentration was observed in the two groups (Table 2). Only 17 patients had significant mitral regurgitation at the end of the SBT: 14 were mild and 3 were moderate. Overall, WiPO was diagnosed in 124/208 (59.6%) and 44/208 (21.2%) patients, according to the liberal and conservative definitions, respectively. WiPO was more frequent in patients who failed the second SBT as compared to successes, and whatever the definition used. WiPO did not influence the overall duration of weaning as assessed by the WIND definition (Table 3). The evolution of other cardiorespiratory parameters during the second SBT is reported in Additional file 2.

**Table 2:** Dynamic changes of biological and echocardiographic data during the second spontaneous breathing trial

Biological and echocardiographic data	Before	At the end	*p*
	Success (*n* = 76)		
Troponin T, ng/l	297 (926)	303 (969)	0.641
NT-proBNP, ng/l	4166 (7316)	3975 (6632)	0.159
Protein, g/l	59.1 (10.9)	60.4 (10.1)	0.040
E mitral wave, cm/s	87.4 (24.4)	89.9 (26.7)	0.248
E/A ratio	0.99 (0.39)	1.00 (0.52)	0.736
E/e’ ratio	10.6 (5.7)	10.7 (5.4)	0.778
LVEF, %	52 (15)	53 (14)	0.323
CO, l/min	5.9 (1.8)	6.2 (2.0)	0.225
	Failure (*n* = 132)		
Troponin T, ng/l	410 (1866)	418 (1921)	0.326
NT-proBNP, ng/l	5726 (14509)	5983 (15476)	0.015
Protein, g/l	59.8 (9.1)	62.0 (9.0)	< 0.001
E mitral wave, cm/s	88.6 (33.7)	101.9 (36.0)	< 0.001
E/A ratio	1.04 (0.54)	1.18 (0.61)	0.003
E/e’ ratio	11.1 (6.7)	11.8 (6.9)	0.040
LVEF, %	54 (14)	55 (15)	0.048
CO, l/min	5.7 (2.0)	6.1 (2.2)	0.012

*NT-proBNP* amino terminal pro-brain natriuretic peptide, *E* early diastolic wave velocity, *A* late diastolic wave velocity, *e’* tissue Doppler early wave velocity at the lateral mitral valve annulus, *LVEF* left ventricle ejection fraction, *CO* cardiac output

Data are expressed as mean (standard deviation). *p* values were calculated using paired Student’s *t* test

**Table 3:** Prevalence of weaning-induced cardiac pulmonary edema (WiPO) and weaning-induced cardiac ischemia (WiCI) during the second spontaneous breathing trial (SBT)

WiPO	All patients (*n* = 208)	Second SBT		*p*	Weaning outcome			*p*
		Success (*n* = 76)	Failure (*n* = 132)		Simple (*n* = 51)	Difficult (*n* = 95)	Prolonged (*n* = 62)	
Liberal definition	124 (59.6)	35 (46.1)	89 (67.4)	0.002	30 (58.8)	54 (56.8)	40 (64.5)	0.626
Conservative definition	44 (21.2)	9 (11.8)	35 (26.5)	0.013	8 (15.7)	22 (23.2)	14 (22.6)	0.524
WiCI	All patients (*n* = 177)	Second SBT		*p*	Weaning outcome			*p*
		Success (*n* = 64)	Failure (*n* = 113)		Simple (*n* = 43)	Difficult (*n* = 83)	Prolonged (*n* = 51)	
ESC 2012	36 (20.3)	13 (20.3)	23 (20.4)	0.995	8 (18.6)	15 (18.1)	13 (25.5)	0.555
AHA 2013	12 (6.8)	3 (4.7)	9 (8.0)	0.540	0 (0.0)	5 (6.0)	7 (13.7)	0.029

*SBT* spontaneous breathing trial, *WiPO* weaning-induced cardiac pulmonary edema, *WiCI* weaning-induced cardiac ischemia, *ESC* European Society of Cardiology, *AHA* American Heart Association

Data are expressed as number of patients (percentage)

WiPO was defined as follows: conservative definition (at least two positive criteria) and liberal definition (at least one positive criterion). Criteria used for WiPO were as follows: (i) echocardiographic findings at the end of the SBT: E/A ratio > 0.95 and E/e’ ratio > 8.5; (ii) increase of BNP (≥ 48 ng/l) or NT-proBNP (≥ 21 ng/l) levels during the SBT; (iii) increase of protein level (> 6%) during the SBT. WiCI was defined as follows: (i) ESC 2012: ST elevation in two contiguous leads (≥ 0.10 mV in all leads other than V_2_–V_3_; ≥ 0.20 mV in V_2_–V_3_ in men ≥ 40 years; ≥ 0.25 mV in V_2_–V_3_ in men < 40 years; ≥ 0.15 mV in V_2_–V_3_ in women), or ST depression ≥ 0.05 mV in two contiguous leads; (ii) AHA 2013: ST elevation or depression ≥ 0.10 mV in two contiguous leads

Weaning outcome was defined as follows: short weaning (successful weaning or death within 1 day after the first SBT), difficult weaning (successful weaning or death after more than 1 day but in less than 7 days after the first SBT), and prolonged weaning (successful weaning or death after 7 days following the first SBT)

**Fig. 1:**
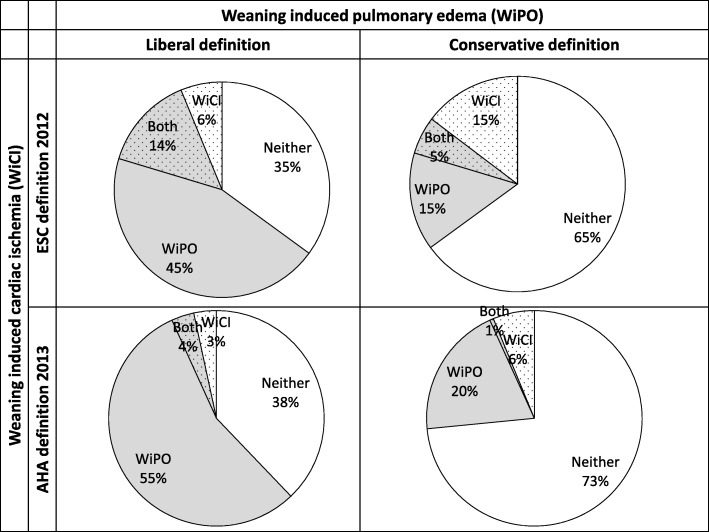
Pie charts of the prevalence of weaning-induced cardiac pulmonary edema (WiPO, according to conservative or liberal definition) and weaning-induced cardiac ischemia (WiCI, according to European Society of Cardiology-ESC-2012 or American Heart Association-AHA-2013 definition) in mechanically ventilated patients during the second spontaneous breathing trial

ST monitoring could be assessed in 177 patients during the second SBT, and WiCI was diagnosed in 36/177 (20.3%) and 12/177 (6.8%) of them, according to the ESC and AHA definitions, respectively. There were more patients admitted for cardiac arrest in the WiCI group as compared to their counterparts (Additional file 3). Changes in troponin were not different between patients with WiCI and their counterparts (Additional file 4). WiCI was not significantly associated with WiPO (Table 4, Fig. 1), neither with the outcome of the second SBT (Table 3). WiCI was more frequent in the prolonged weaning group when using the AHA, but not ESC definition (Table 3).

**Table 4:** Prevalence of weaning-induced cardiac pulmonary edema (WiPO) in patients with weaning-induced cardiac ischemia (WiCI) during the second spontaneous breathing trial

WiCI	WiPO (liberal definition)		*p*	WiPO (conservative definition)		*p*
	No (*n* = 73)	Yes (*n* = 104)		No (*n* = 141)	Yes (*n* = 36)	
ESC 2012	11 (15.1)	25 (24.0)	0.144	26 (18.4)	10 (27.8)	0.214
AHA 2013	6 (8.2)	6 (5.8)	0.555	11 (7.8)	1 (2.8)	0.464

*WiPO* weaning-induced cardiac pulmonary edema, *WiCI* weaning-induced cardiac ischemia, *ESC* European Society of Cardiology, *AHA* American Heart Association

Data are expressed as number of patients (percentage)

WiPO was defined as follows: conservative definition (at least two positive criteria) and liberal definition (at least one positive criterion). Criteria used for WiPO were as follows: (i) echocardiographic findings at the end of the SBT: E/A ratio > 0.95 and E/e’ ratio > 8.5; (ii) increase of BNP (≥ 48 ng/l) or NT-proBNP (≥ 21 ng/l) levels during the SBT; (iii) increase of protein level (> 6%) during the SBT. WiCI was defined as follows: (i) ESC 2012: ST elevation in two contiguous leads (≥ 0.10 mV in all leads other than V_2_–V_3_; ≥ 0.20 mV in V_2_–V_3_ in men ≥ 40 years; ≥ 0.25 mV in V_2_–V_3_ in men < 40 years; ≥ 0.15 mV in V_2_–V_3_ in women), or ST depression ≥ 0.05 mV in two contiguous leads; (ii) AHA 2013: ST elevation or depression ≥ 0.10 mV in two contiguous leads

### Coronary exploration or treatment

Two patients of the cohort were treated for an acute coronary syndrome after the second SBT, including one patient with a percutaneous reperfusion (the other patient presented a hemorrhagic shock which precluded any reperfusion therapy). Seven patients with a past medical history of coronary artery disease failed their second SBT and were explored with coronary angiography: two patients had coronary lesions without necessity of reperfusion therapy while five patients had no significant coronary lesions (Additional file 5).

## Discussion

We herein report the largest cohort of ventilated patients who were explored for WiCI and WiPO during weaning, using multiple noninvasive tools including echocardiography, ST monitoring, and biomarkers. The prevalence of WiPO and that of WiCI were not negligible, but their association was weak. In addition, their impact on weaning outcomes was not consistent and depended on the definition and outcome used.

### WiPO

WiPO is one of the main mechanisms of weaning failure [5]. We found a high prevalence of WiPO after a first SBT failure. This result is consistent with previous studies [8, 17, 25, 26]. The gold standard to evaluate LV filling pressures is to measure the pulmonary artery occlusion pressure using a pulmonary artery catheter. Because our patients were not equipped with such device, we chose to combine three noninvasive criteria as proposed by the recent literature, namely echocardiography [17], cardiac biomarkers [9], and protidemia [8]. We observed significant dynamic changes in these surrogates of left atrial pressure during SBT failure, and the prevalence of WiPO in our study (21 to 60%) is in accordance with that found with the gold standard pulmonary artery catheter (44%) [17].

Overall, WiPO was more frequent in patients who failed the second SBT as compared to successes, whatever the definition used (conservative or liberal). However, nearly half of the patients who succeeded the second SBT had at least one criterion for WiPO. This result is consistent with previous reports suggesting a significant increase in left atrial pressure during SBT, even in the case of success [27].

The lack of association between WiPO and weaning outcomes in our series may be explained by an early depletive management prompted by the initial evolution and the first SBT failure. In fact, more than half of patients received diuretics before the second SBT. Patient selection may also be important. Only one quarter of our patients had a short weaning (with a successful extubation less than 24 h after the first SBT), a proportion lower than that reported in the WIND study (57%). This discrepancy is at least in part explained by the fact that we included only patients who failed their first SBT. The incidence of WIPO in our study could be overestimated due to the increased proportion of unsuccessful SBT after a first SBT failure. The median delay between the first and the second SBT was very short in our study with a median of 1 day. These patients were successfully extubated on the next day (within 24 h after the first and the second SBT).

### WiCI

Prevalence of WiCI during the second SBT was less pronounced, as compared to WiPO, and varied between 6.8% and 20.3%, depending on the definition used. During weaning, silent myocardial ischemia was reported in high-risk postoperative patients [28] and in patients with a known coronary artery disease [23]. Prevalence of WiCI in the general population of critically ill patients varied between 0 and 70% across small cohorts [11, 24, 29], but the method of diagnosis and definitions often differed from ours. The association between WiCI and weaning outcome was weak in our study and only significant for the most restrictive definition derived from AHA statement for exercise testing. Electrocardiographic changes may not be specific to diagnose myocardial ischemia and may reflect myocardial injury whatever its cause [30]. However, ECG monitoring during cardiac stress is recommended as a first-line test to diagnose stable coronary artery disease and to detect transient myocardial ischemia [22, 31]. The association between WiCI and WiPO was also weak, suggesting that non-ischemic mechanisms may instead contribute to WiPO. In fact, the role of diastolic dysfunction seems central in the pathophysiology of WiPO [18]. The removal of positive-pressure support during weaning increases left ventricular preload and afterload and may impair left ventricular compliance [32, 33]. The ability of left ventricle to improve diastolic performance and maintain normal filling pressures during stress may be of paramount importance during weaning. Future strategies should aim at differentiating non-ischemic myocardial injury from myocardial infarction [30]. Biomarkers of cardiac injury (troponin) seemed of little help to detect transient myocardial ischemia during weaning in our study. Only three patients had significant variations in troponin I during weaning, and none of these patients experienced electrocardiographic changes. Further studies should assess the usefulness of other biomarkers with a shorter half-life time (e.g., myoglobin) in this setting.

### Therapeutic implications

Only two patients with significant modifications of ST monitoring were treated for an acute coronary syndrome after the second SBT. Although quite infrequent, aggressive therapeutic interventions (such as coronary reperfusion) may sometimes be required to successfully extubate those patients [34, 35]. The pre-existence of a known coronary artery disease (before admission to ICU) was not associated with WiCI. Whether undiagnosed coronary artery disease could be associated with WiCI remains unknown. Future studies should explore strategies aimed at detecting patients with clinically relevant cardiac ischemia during the weaning process.

### Strengths and limitations

Out study is the first to explore myocardial ischemia in a large cohort of patients who failed a first SBT. Strengths of our study include its prospective and multicentric design and the comprehensive assessment of cardiac function with multiple tools (echocardiography, continuous 12-lead ECG ST measurement, cardiac biomarkers). Limitations include the non-blinded and observational nature of the study, the absence of invasive monitoring which precluded any direct measurement of left atrial pressure, and the need to use multiple indirect criteria to define WiPO, owing to the lack of consensus in the literature. We did not assess lung ultrasonography because studies suggesting a role for this technique for the assessment of WIPO [36] were published after we started our study. In addition, the generalizability of our findings is limited by the fact that we examined a sub-sample of patients who had failed an initial SBT. Last, mitral regurgitation assessment was not exhaustive in our study and mainly used color Doppler, which may have led to quantification errors.

## Conclusions

WiPO occurred in a significant number of critically ill patients who failed a first SBT, while WiCI was less frequent. The correlation between WiPO and WiCI was weak, and their association with weaning outcomes was weak in this non-blinded observational series.

## Supplementary information

**Additional file 1.** Characteristics and outcomes of 208 patients during weaning. (PDF 113 kb)

**Additional file 2.** Clinical and biological variables during the second spontaneous breathing trial (SBT). (PDF 145 kb)

**Additional file 3.** Characteristics and outcomes of 208 patients who failed a first spontaneous breathing trial (SBT), according to the prevalence of weaning-induced cardiac ischemia (WiCI). (PDF 147 kb)

**Additional file 4.** Changes in biological variables during the second spontaneous breathing trial (SBT), according to the prevalence of weaning-induced cardiac ischemia (WiCI). (PDF 131 kb)

**Additional file 5.** Characteristics of nine patients with coronary invasive exploration or treatment during weaning. (PDF 135 kb)

## Data Availability

The datasets used and/or analyzed during the current study are available from the corresponding author on reasonable request.

## Abbreviations

A: Late diastolic mitral wave velocity
a’: Tissue Doppler late diastolic wave velocity at the lateral mitral valve annulus
AHA: American Heart Association
BNP: Brain natriuretic peptide
E: Early diastolic mitral wave velocity
e’: Tissue Doppler early diastolic wave velocity at the lateral mitral valve annulus
ESC: European Society of Cardiology
FiO_2_: Fraction of inspired oxygen
NT-proBNP: Amino terminal pro-brain natriuretic peptide
PEEP: Positive end-expiratory pressure
SBT: Spontaneous breathing trial
WiPO: Weaning-induced pulmonary edema
WiCI: Weaning-induced cardiac ischemia
