# Supplementary material for: Myocardial ischemia during ventilator weaning: a prospective multicenter cohort study
Source: Crit Care. 2019 Sep 18;23:321. doi: 10.1186/s13054-019-2601-8 (PMC6751853; doi:10.1186/s13054-019-2601-8)
Supplement: Supplementary file 1 — Additional file 1. Characteristics and outcomes of 208 patients during weaning. (PDF 113 kb) [file 13054_2019_2601_MOESM1_ESM.pdf]

Additional file 1. Characteristics and outcomes of 208 patients during weaning

	All patients	Weaning outcome			
		Short	Difficult	Prolonged	
	(n=208)	(n=51)	(n=95)	(n=62)	p
Age, year	67 (56-75)	65 (52-72)	64 (56-75)	70 (62-76)	0.075
Female sex	76 (36.5)	17 (33.3)	37 (38.9)	22 (35.5)	0.781
Body mass index, kg/m^2	27 (22-32)	27 (23-30)	28 (22-33)	26 (23-32)	0.883
SAPS-II at admission	50 (39-63)	51 (34-59)	50 (40-65)	48 (40-62)	0.528
MacCabe score					0.524
0	120 (57.7)	32 (62.7)	58 (61.1)	30 (48.4)	
1	57 (27.4)	12 (23.5)	24 (25.3)	21 (33.9)	
2	31 (14.9)	7 (13.7)	13 (13.7)	11 (17.7)	
Comorbidities					
COPD	52 (25.0)	9 (17.6)	24 (25.3)	19 (30.6)	0.283
Restrictive lung disease	23 (11.1)	4 (7.8)	11 (11.6)	8 (12.9)	0.678
Obstructive sleep apnoea syndrome	26 (12.5)	10 (19.6)	11 (11.6)	5 (8.1)	0.170
Asthma	7 (3.4)	4 (7.8)	1 (1.1)	2 (3.2)	0.095
Current smoker	94 (45.2)	22 (43.1)	44 (46.3)	28 (45.2)	0.935
Quantification of cigarette smoking, pack-year	34 (20-50)	45 (31-66)	31 (20-45)	31 (20-52)	0.268
Central nervous system disease	19 (9.1)	3 (5.9)	9 (9.5)	7 (11.3)	0.603
Peripheral neuropathy	14 (6.7)	4 (5.9)	8 (8.4)	3 (4.8)	0.656
Mental illness	11 (5.3)	4 (7.8)	5 (5.3)	2 (3.2)	0.551
Heart failure with preserved ejection fraction	33 (15.9)	14 (27.5)	13 (13.7)	6 (9.7)	0.027
Heart failure with reduced ejection fraction	34 (16.3)	4 (7.8)	13 (13.7)	17 (27.4)	0.013
Atrial fibrillation	42 (20.2)	7 (13.7)	21 (22.1)	14 (22.6)	0.415
Hypertension	111 (53.4)	29 (56.9)	51 (53.7)	31 (50.0)	0.765
Valvular heart disease	24 (11.5)	9 (17.6)	10 (10.5)	5 (8.1)	0.260
Coronary artery disease	41 (19.7)	12 (23.5)	14 (14.7)	15 (24.2)	0.254
Pulmonary hypertension	12 (5.8)	3 (5.9)	7 (7.4)	2 (3.2)	0.553
Reason for intubation					
Coma	28 (13.5)	9 (17.6)	11 (11.6)	8 (12.9)	0.585
Septic shock	31 (14.9)	8 (15.7)	11 (11.6)	12 (19.4)	0.402
COPD exacerbation	19 (9.1)	6 (11.8)	9 (9.5)	4 (6.5)	0.703
Pneumonia	59 (28.4)	11 (21.6)	33 (34.7)	15 (24.2)	0.166
Cardiogenic pulmonary edema	20 (9.6)	8 (15.7)	8 (8.4)	4 (6.5)	0.220
Cardiac arrest	20 (9.6)	3 (5.9)	9 (9.5)	8 (12.9)	0.451
Surgery	15 (7.2)	2 (3.9)	8 (8.4)	5 (8.1)	0.577
Others	15 (7.2)	4 (7.8)	5 (5.3)	6 (9.7)	0.568
Events between ICU admission and inclusion					
Acute respiratory distress syndrome	87 (41.8)	18 (35.3)	38 (40.0)	31 (50.0)	0.256
Septic shock	107 (51.4)	21 (41.2)	46 (48.4)	40 (64.5)	0.034
Ventilator-associated pneumonia	53 (25.5)	8 (15.7)	19 (20.0)	26 (41.9)	0.002
Neuromuscular blockade	100 (48.5)	19 (37.3)	44 (47.3)	37 (59.7)	0.057
Atrial fibrillation	71 (34.1)	14 (27.5)	30 (31.6)	27 (43.5)	0.155
Corticosteroids	67 (32.3)	14 (27.5)	32 (33.7)	21 (33.9)	0.704
Delay between admission and inclusion, days	7 (4-14)	6 (3-9)	6 (3-9)	14 (7-19)	<0.001
Prophylactic NIV after extubation	126 (60.6)	34 (66.7)	62 (65.3)	30 (48.4)	0.063
Outcomes					
Successful weaning[a]	176 (84.6)	51 (100.0)	90 (94.7)	35 (56.5)	<0.001
Tracheotomy	8 (3.8)	0	0	8 (12.9)	<0.001
Ventilator-free days at day-28, days[b]	16 (0-22)	22 (19-25)	20 (14-22)	0 (0-0)	<0.001
Length of stay in ICU, days	14 (9-24)	10 (6-14)	13 (10-17)	33 (29-45)	<0.001
Death in ICU	44 (21.2)	0 (0.0)	9 (9.5)	35 (56.5)	<0.001

SAPS Simplified Acute Physiologic score, COPD chronic obstructive pulmonary disease, ICU intensive care unit, NIV non invasive ventilation

Data are expressed as number (percentage) for categorical variables or median (1st quartile- 3rd quartile) for continuous variables.

Weaning outcome was defined as follows: short weaning (successful weaning or death within one day after the first SBT), difficult weaning (successful weaning or death after more than one day but in less than seven days after the first SBT), and prolonged weaning (successful weaning or death after seven days following the first SBT).

[a] Successful weaning was defined as patient alive and extubated within the next 7 days after extubation; [b] If patient died before day-28, VFD at day-28=0
