# Supplementary material for: Myocardial ischemia during ventilator weaning: a prospective multicenter cohort study
Source: Crit Care. 2019 Sep 18;23:321. doi: 10.1186/s13054-019-2601-8 (PMC6751853; doi:10.1186/s13054-019-2601-8)
Supplement: Supplementary file 2 — Additional file 2. Clinical and biological variables during the second spontaneous breathing trial (SBT). (PDF 145 kb) [file 13054_2019_2601_MOESM2_ESM.pdf]

Additional file 2. Clinical and biological variables during the second spontaneous breathing trial (SBT)

	All patients	Second SBT		
		Success	Failure	
	(n=208)	(n=76)	(n=132)	p
Clinical variables				
Respiratory rate at the beginning	27 (22-31)	26 (21-31)	27 (23-32)	0.108
Respiratory rate at the end	34 (28-40)	29 (24-33)	39 (32-42)	<0.001
SpO_2 at the beginning, %	97 (95-98)	97 (95-98)	96 (94-98)	0.011
SpO_2 at the end, %	94 (91-97)	96 (93-98)	93 (89-96)	<0.001
Heart rate at the beginning, beats/min	91 (80-105)	91 (81-105)	90 (79-104)	0.501
Heart rate at the end, beats/min	99 (85-112)	97 (82-108)	100 (87-114)	0.145
Systolic blood pressure at the beginning, mmHg	134 (121-150)	134 (124-150)	135 (119-150)	0.495
Systolic blood pressure at the end, mmHg	141 (125-163)	136 (122-150)	148 (126-173)	0.002
Biological variables				
PaO_2/FiO_2 at the beginning	247 (207-302)	252 (215-303)	241 (202-300)	0.185
PaO_2/FiO_2 at the end	168 (121-214)	184 (142-222)	165 (114-206)	0.020
$PaCO_2$ at the beginning, mmHg	40 (34-46)	38 (33-43)	41 (36-47)	0.003
$PaCO_2$ at the end, mmHg	42 (36-49)	39 (35-47)	43 (37-52)	0.017
HCO_3^- at the beginning, mmol/l	29 (25-32)	28 (25-31)	29 (25-33)	0.167
HCO_3^- at the end, mmol/l	29 (25-33)	29 (25-31)	29 (26-34)	0.293
Lactate at the beginning, mmol/l	0.9 (0.7-1.2)	1.0 (0.7-1.4)	0.9 (0.7-1.2)	0.342
Lactate at the end, mmol/l	1.0 (0.6-1.4)	0.8 (0.7-1.3)	1 (0.6-1.4)	0.464
Troponin T at the beginning, ng/l	53 (23-134)	47 (21-138)	54 (24-134)	0.497
Troponin T at the end, ng/l	52 (24-151)	47 (21-165)	53 (25-147)	0.546
(Troponin T at the beginning <14 ng/ml and Δ Troponin T > 50%) or (Troponin T ≥ 14 ng/ml and Δ Troponin T > 20%) or Δ Troponin I > 20%	3 (1.4)	1 (1.3)	2 (1.5)	>0.99
NTpro-BNP at the beginning, ng/l	1089 (379-4032)	1356 (293-3856)	1077 (420-4058)	0.790
NTpro-BNP at the end, ng/l	1111 (438-4034)	1111 (321-3914)	1090 (463-4182)	0.679
Protein at the beginning, g/l	59 (54-66)	58 (53-66)	60 (54-65)	0.625
Protein at the end, g/l	61 (56-68)	60 (53-68)	61 (57-67)	0.395

SBT Spontaneous breathing trial, NTpro-BNP amino terminal pro-brain natriuretic peptide

Data are expressed as number (percentage) for categorical variables or median (1st quartile- 3rd quartile) for continuous variables.
