# Supplementary material for: Myocardial ischemia during ventilator weaning: a prospective multicenter cohort study
Source: Crit Care. 2019 Sep 18;23:321. doi: 10.1186/s13054-019-2601-8 (PMC6751853; doi:10.1186/s13054-019-2601-8)
Supplement: Supplementary file 3 — Additional file 3. Characteristics and outcomes of 208 patients who failed a first spontaneous breathing trial (SBT), according to the prevalence of weaning-induced cardiac ischemia (WiCI). (PDF 147 kb) [file 13054_2019_2601_MOESM3_ESM.pdf]

**Additional file 3. Characteristics and outcomes of 208 patients who failed a first spontaneous breathing trial (SBT), according to the prevalence of weaning-induced cardiac ischemia (WiCI)**

| | WiCI | | |
|---|---|---|---|
| | No (n=141) | Yes (n=36) | p |
| Age, year | 66 (56-73) | 70 (60-77) | 0.147 |
| Female sex | 48 (34.0) | 18 (50.0) | 0.077 |
| Body mass index, kg/$m^2$ | 27 (22-32) | 26 (23-30) | 0.669 |
| SAPS-II at admission | 50 (40-63) | 50 (35-64) | 0.702 |
| MacCabe score | | | 0.606 |
| 0 | 75 (53.2) | 22 (61.1) | |
| 1 | 42 (29.8) | 10 (27..8) | |
| 2 | 24 (17.0) | 4 (11.1) | |
| *Comorbidities* | | | |
| COPD | 41 (29.1) | 5 (13.9) | 0.064 |
| Restrictive lung disease | 16 (11.3) | 6 (16.7) | 0.400 |
| Obstructive sleep apnea syndrome | 21 (14.9) | 4 (11.1) | 0.561 |
| Asthma | 2 (1.4) | 0 (0.0) | >0.99 |
| Current smoker | 67 (47.5) | 14 (38.9) | 0.354 |
| Quantification of cigarette smoking, pack-year | 40 (26-55) | 30 (20-50) | 0.406 |
| Central nervous system disease | 15 (10.6) | 4 (11.1) | >0.99 |
| Peripheral neuropathy | 11 (7.8) | 1 (2.8) | 0.464 |
| Mental illness | 8 (5.7) | 1 (2.8) | 0.688 |
| Heart failure with preserved ejection fraction | 21 (14.9) | 4 (11.1) | 0.561 |
| Heart failure with reduced ejection fraction | 26 (18.8) | 2 (5.6) | 0.059 |
| Atrial fibrillation | 26 (18.4) | 9 (25.0) | 0.378 |
| Hypertension | 71 (50.4) | 24 (66.7) | 0.080 |
| Valvular heart disease | 17 (12.1) | 4 (11.1) | >0.99 |
| Coronary disease | 29 (20.6) | 7 (19.4) | 0.881 |
| Pulmonary hypertension | 12 (8.5) | 0 (0.0) | 0.129 |
| *Reason for intubation* | | | |
| Coma | 20 (14.2) | 4 (11.1) | 0.788 |
| Septic shock | 25 (17.7) | 4 (11.1) | 0.338 |
| COPD exacerbation | 14 (9.9) | 2 (5.6) | 0.624 |
| Pneumonia | 39 (27.7) | 8 (22.2) | 0.510 |
| Cardiogenic pulmonary edema | 13 (9.2) | 4 (11.1) | 0.731 |
| Cardiac arrest | 8 (5.7) | 8 (22.2) | 0.005 |
| Surgery | 10 (7.1) | 4 (11.1) | 0.489 |
| Others | 11 (7.8) | 2 (5.6) | >0.99 |
| *Events between ICU admission and inclusion* | | | |
| Acute respiratory distress syndrome | 62 (44.0) | 10 (27.8) | 0.078 |
| Septic shock | 76 (53.9) | 15 (38.9) | 0.108 |
| Ventilator-associated pneumonia | 34 (24.1) | 10 (27.8) | 0.650 |
| Neuromuscular blockade | 69 (49.3) | 15 (42.9) | 0.496 |
| Atrial fibrillation | 45 (31.9) | 14 (38.9) | 0.428 |
| Corticosteroids | 51 (36.2) | 8 (22.2) | 0.113 |
| Time between admission and inclusion, days | 7 (4-14) | 6 (4-10) | 0.614 |
| *Outcomes* | | | |
| Success weaning (within 7 days) | 116 (82.3) | 33 (91.7) | 0.168 |
| Tracheotomy | 7 (5.0) | 1 (2.8) | >0.99 |
| Ventilator-free days at day-28, days | 16 (0-21) | 18 (3-22) | 0.272 |
| Length of stay in ICU, days | 14 (10-23) | 12 (9-27) | 0.838 |
| Death in ICU | 33 (23.4) | 5 (13.9) | 0.215 |

*SBT Spontaneous breathing trial, SAPS Simplified Acute Physiologic score, COPD chronic obstructive pulmonary disease, ICU intensive care unit*

*Data are expressed as number (percentage) for categorical variables or median (1st quartile- 3rd quartile) for continuous variables.*

WiCI was defined as follows: i) ESC 2012: ST elevation in two contiguous leads (≥0.10 mV in all leads other than $V_2$-$V_3$; ≥0.20 mV in $V_2$-$V_3$ in men ≥40 years; ≥0.25 mV in $V_2$-$V_3$ in men <40 years; ≥0.15 mV in $V_2$-$V_3$ in women), or ST depression ≥0.05 mV in two contiguous leads; ii) AHA 2013: ST elevation or depression ≥0.10 mV in two contiguous leads.

[a] Successful weaning was defined as patient alive and extubated within the next 7 days after extubation; [b] If patient died before day-28, VFD at day-28=0
