# Supplementary material for: Myocardial ischemia during ventilator weaning: a prospective multicenter cohort study
Source: Crit Care. 2019 Sep 18;23:321. doi: 10.1186/s13054-019-2601-8 (PMC6751853; doi:10.1186/s13054-019-2601-8)
Supplement: Supplementary file 4 — Additional file 4. Changes in biological variables during the second spontaneous breathing trial (SBT), according to the prevalence of weaning-induced cardiac ischemia (WiCI). (PDF 131 kb) [file 13054_2019_2601_MOESM4_ESM.pdf]

Additional file 4. Changes in biological variables during the second spontaneous breathing trial (SBT), according to the prevalence of weaning-induced cardiac ischemia (WiCI).

	WiCI		
	No (n=141)	Yes (n=36)	p
Troponin T at the beginning, ng/l	56 (25-126)	50 (23-220)	0.938
Troponin T at the end, ng/l	54 (26-136)	50 (22-224)	0.994
NTpro-BNP at the beginning, ng/l	1069 (401-3941)	1425 (266-3308)	0.753
NTpro-BNP at the end, ng/l	1056 (467-4175)	1407 (266-3302)	0.706
Protein at the beginning, g/l	60 (54-67)	57 (53-63)	0.124
Protein at the end, g/l	61 (56-68)	60 (57-68)	0.946

NTpro-BNP amino terminal pro-brain natriuretic peptide

Data are expressed as median (1st quartile- 3rd quartile). p-values were calculated using the Mann-Whitney U test.

WiCI was defined as follows: i) ESC 2012: ST elevation in two contiguous leads (≥0.10 mV in all leads other than V_2-V_3; ≥0.20 mV in V_2-V_3 in men ≥40 years; ≥0.25 mV in V_2-V_3 in men <40 years; ≥0.15 mV in V_2-V_3 in women), or ST depression ≥0.05 mV in two contiguous leads; ii) AHA 2013: ST elevation or depression ≥0.10 mV in two contiguous leads.
