# Supplementary material for: Myocardial ischemia during ventilator weaning: a prospective multicenter cohort study
Source: Crit Care. 2019 Sep 18;23:321. doi: 10.1186/s13054-019-2601-8 (PMC6751853; doi:10.1186/s13054-019-2601-8)
Supplement: Supplementary file 5 — Additional file 5. Characteristics of nine patients with coronary invasive exploration or treatment during weaning. (PDF 135 kb) [file 13054_2019_2601_MOESM5_ESM.pdf]

Additional file 5. Characteristics of nine patients with coronary invasive exploration or treatment during weaning

	SBT outcome	WiCI	WiPO	Coronary angiography	Treatment
Patient #1	Failure	No	Yes	Normal	None
Patient #2	Failure	No	No	Normal	Medical
Patient #3	Failure	No	Yes	Normal	Medical
Patient #4	Failure	No	Yes	Normal	None
Patient #5	Failure	No	Yes	CAD	Medical
Patient #6	Failure	No	Yes	CAD	Medical
Patient #7	Failure	No	Yes	Normal	None
Patient #8	Failure	Yes	Yes	CAD	Angioplasty
Patient #9	Failure	Yes	Yes	Not done	Medical

SBT Spontaneous breathing trial, CAD Coronary artery disease

WiPO was defined as follows: conservative definition (at least 2 positive criteria), and liberal definition (at least 1 positive criterion). Criteria used for WiPO were: i) echocardiographic findings at the end of the SBT: E/A ratio >0.95 and E/e' ratio >8.5; ii) increase of BNP (≥48 ng/l) or NT-proBNP (≥21 ng/l) levels during the SBT; iii) increase of protein level (>6 %) during the SBT. WiCI was defined as follows: i) ESC 2012: ST elevation in two contiguous leads (≥0.10 mV in all leads other than V_2-V_3; ≥0.20 mV in V_2-V_3 in men ≥40 years; ≥0.25 mV in V_2-V_3 in men <40 years; ≥0.15 mV in V_2-V_3 in women), or ST depression ≥0.05 mV in two contiguous leads; ii) AHA 2013: ST elevation or depression ≥0.10 mV in two contiguous leads
